# Developing a machine learning-based predictive model for levothyroxine dosage estimation in hypothyroid patients: a retrospective study

**DOI:** 10.3389/fendo.2025.1415206

**Published:** 2025-03-14

**Authors:** Tran Thi Ngan, Dang Huong Tra, Ngo Thi Quynh Mai, Hoang Van Dung, Nguyen Van Khai, Pham Van Linh, Nguyen Thi Thu Phuong

**Affiliations:** ^1^ Faculty of Pharmacy & Biomedical-Pharmaceutical Sciences Research Group, Hai Phong University of Medicine and Pharmacy, Hai Phong, Vietnam; ^2^ Pharmacy Department, Hai Phong International Hospital, Hai Phong, Vietnam; ^3^ Department of Rheumatology-Nephrology-Allergy and Immunology, Hai Phong International Hospital, Hai Phong, Vietnam; ^4^ Faculty of Public Health, Hai Phong University of Medicine and Pharmacy, Hai Phong, Vietnam; ^5^ Faculty of Medicine, Hai Phong University of Medicine and Pharmacy, Hai Phong, Vietnam

**Keywords:** levothyroxine, hypothyroidism, model estimation, endocrine, retrospective study

## Abstract

Hypothyroidism, a common endocrine disorder, has a high incidence in women and increases with age. Levothyroxine (LT4) is the standard therapy; however, achieving clinical and biochemical euthyroidism is challenging. Therefore, developing an accurate model for predicting LT4 dosage is crucial. This retrospective study aimed to identify factors affecting the daily dose of LT4 and develop a model to estimate the dose of LT4 in hypothyroidism from a cohort of 1,864 patients through a comprehensive analysis of electronic medical records. Univariate analysis was conducted to explore the relationships between clinical and non-clinical variables, including weight, sex, age, body mass index, diastolic blood pressure, comorbidities, food effects, drug-drug interactions, liver function, serum albumin and TSH levels. Among the models tested, the Extra Trees Regressor (ETR) demonstrated the highest predictive accuracy, achieving an R² of 87.37% and the lowest mean absolute error of 9.4 mcg (95% CI: 7.7–11.2) in the test set. Other ensemble models, including Random Forest and Gradient Boosting, also showed strong performance (R² > 80%). Feature importance analysis highlighted BMI (0.516 ± 0.015) as the most influential predictor, followed by comorbidities (0.120 ± 0.010) and age (0.080 ± 0.005). The findings underscore the potential of machine learning in refining LT4 dose estimation by incorporating diverse clinical factors beyond traditional weight-based approaches. The model provides a solid foundation for personalized LT4 dosing, which can enhance treatment precision and reduce the risk of under- or over-medication. Further validation in external cohorts is essential to confirm its clinical applicability.

## 1 Introduction

Hypothyroidism, a prevalent endocrine disorder, exhibits varying incidence and prevalence across different demographic groups (1). With a prevalence tenfold higher in women than in men, its occurrence escalates with advancing age. Hashimoto’s thyroiditis is the leading cause of hypothyroidism in iodine-sufficient areas, affecting 20-30% of patients (2). It is part of a spectrum of autoimmune thyroid disorders with a complex pathogenesis involving genetic susceptibility and environmental factors (3). The disease is characterized by lymphocytic infiltration and follicular destruction, leading to thyroid atrophy and fibrosis (2, 3). Both cellular and humoral immunity play crucial roles, with defects in T regulatory cells and increased activation of follicular helper T cells contributing to disease initiation and perpetuation. Recent studies have identified multiple cytokine networks involving thyroid cells in proinflammatory effects and various T regulatory cell defects underlying the loss of self-tolerance (4). Environmental factors, including improved hygiene, increased dietary iodine intake, and certain medications, have been implicated in the recent increase in HT incidence (3). Diagnosis primarily relies on a combination of clinical and biochemical assessments due to the nonspecific nature of symptoms. Thyroid hormone replacement therapy has been used to treat hypothyroidism for over a century (5). Levothyroxine (LT4) the standard therapy for hypothyroid patients affecting approximately 5% of the global population, has significantly enhanced the quality of life for millions since its inception in 1949. Nonetheless, ensuring consistent biochemical and clinical euthyroidism in LT4-treated individuals remains a significant challenge (6). Given that LT4 is typically administered lifelong, changes in physiology necessitate adjustments in dosage to maintain euthyroidism (6). Moreover, dose modifications may be imperative for patients with concurrent medical conditions, those receiving specific medications, and elderly patients. Individuals undergoing weight fluctuations or hormonal changes may also necessitate dose adjustments, with pregnant women often requiring increased LT4 doses. Achieving the best treatment outcomes for hypothyroidism involves a collaborative effort between the patient and the physician. The physician’s role entails evaluating the patient’s condition through comprehensive clinical and laboratory assessments, and making necessary adjustments to LT4 therapy accordingly (6).

Drugs can change thyroid function in individuals without a thyroid illness, with effects ranging from aberrant thyroid biochemical parameters to overt thyroid malfunctions. The same medications may change the LT4 needs of patients currently being treated for hypothyroidism (7–9). These changes are exemplified by estrogen (10) and androgens (11), potentially causing increased and decreased LT4 requirements, respectively. Substances such as calcium and iron are prone to binding to LT4 in the gastrointestinal tract, potentially diminishing its absorption. Similarly, proton pump inhibitors may hinder absorption by elevating gastric pH (12). Methimazole, propylthiouracil, checkpoint inhibitors, alemtuzumab, interferon-α, amiodarone, sunitinib, and lithium are among the medications that could be included in this category due to their impact on thyroid hormone synthesis or release (13). Normalization of serum thyroid-stimulating hormone (TSH) levels before pregnancy is critical in patients with hypothyroidism (14). Early in the first trimester of pregnancy, women treated for hypothyroidism often require a 20%–30% increase in their LT4 dose. This increased demand is caused by mechanisms such as increased hepatic thyroxine-binding globulin (TBG) production and thyroid hormone metabolism by placental type 3 deiodinase (15, 16). Multiple studies have indicated a reduction in the required LT4 dose among elderly people (17–19). However, a recent study has proposed that this decline in LT4 requirement may be attributed to age-related changes in weight (20). Factors such as body weight, ideal body weight, and lean body mass all play a role in determining the necessary LT4 dosage, with higher levels of these factors correlating with increased dosage requirements (17, 21). However, if actual body weight is utilized to determine the dosage of LT4 needed by people with obesity, the dose may be overestimated instead of being more accurately predicted by optimum body weight (21). The use of LT4 for the treatment of thyroid disorders has been well established. However, recent studies have shown that this medication’s dosage can significantly affect the liver (22–24). Appropriate LT4 dosage adjustment is crucial for preventing liver injury. In some cases, it may be necessary to base the dosage adjustment on the serum levels of glutamic oxaloacetic transaminase (GOT) and glutamic pyruvic transaminase (GPT). As noted by Silva et al. (25), individuals with elevated GPT levels may have a higher risk of abdominal circumference and total cholesterol.

The practice of initiating dosing based on weight is thought to be insufficient, as approximately 70% of patients often necessitate dosage adjustments during their initial postoperative follow-up (26). Additionally, administering an excessive LT4 dosage elevates the risk of accelerated bone loss, fractures, heat intolerance, diarrhea, and arrhythmias (27, 28). Conversely, underdosing of LT4 dosing leads to symptoms of hypothyroidism, such as fatigue and weight gain (29–31). Acknowledging these challenges, our study aims to systematically investigate the factors influencing LT4 dosage and develop a predictive model that incorporates the diverse determinants of dose variability. This model is intended to accurately estimate the appropriate levothyroxine dose, optimize therapeutic precision, and improve patient outcomes in the management of hypothyroidism.

## 2 Materials and methods

Our study, conducted in 2024, included data collected from the electronic medical records of outpatients at Hai Phong International Hospital between January 2022 and December 2023 who met the following criteria:

### 2.1 Participants

#### 2.1.1 Selection criteria

- Patients diagnosed with hypothyroidism based on ICD-10 codes, including E02, E03, E89.0.
- Patients meeting diagnostic criteria for hypothyroidism, including overt hypothyroidism (elevated TSH levels with low free thyroxine levels) and subclinical hypothyroidism (elevated TSH levels with normal free thyroxine levels).
- Patients prescribed LT4 therapy at the study site.
D Patients with documented monthly follow-up visits at the study site for at least three consecutive months.

#### 2.1.2 Exclusion criteria

- Use of LT4 outside the study site.
- Missing TSH test results.
- Incomplete medical records lacking clinical information.

Hypothyroidism is diagnosed based on elevated levels of TSH and reduced levels of free thyroxine (32). The normal reference range for free thyroxine levels is approximately 12 to 22 pmol/L. The normal reference range of serum TSH is within approximately 0.5–5.0 mU/L (17, 33).

#### 2.1.3 Drug study

All patients were administered LT4 at 50 mcg or 100 mcg doses.

#### 2.1.4 Data processing

During the study period, 3,794 medical records of patients diagnosed with hypothyroidism were reviewed and screened. The data extraction process involved accessing the hospital’s electronic medical record (EMR) system, which stores comprehensive patient information. A systematic query was performed using predefined criteria to identify patients diagnosed with hypothyroidism and prescribed LT4 between January 2022 and December 2023. The query extracted relevant data fields, including demographic information (e.g., age, sex, weight), clinical details (e.g., symptoms, comorbid conditions), laboratory results (e.g., TSH levels, free T4 levels), and prescription records.

Data cleaning and verification were then conducted to ensure accuracy and completeness. Records were excluded if they indicated LT4 use outside the study site, if follow-up visits were inconsistent (less than three months), or if key information, such as TSH test results or clinical history, was missing. Specifically:

- 788 records were excluded for LT4 use outside the study site or insufficient follow-up.
- 328 records were excluded due to incomplete laboratory or clinical data.

This resulted in a final dataset of 2,678 medical records for analysis. Within this cohort, 69.6% (1,864 patients) achieved the hypothyroidism treatment goal after three months of follow-up, defined as symptom resolution and normalization of serum TSH levels within the reference range of 0.5–5.0 mU/L.

The extracted data was then anonymized to maintain patient confidentiality and imported into a secure database for further statistical analysis.

### 2.2 Statistical analysis

#### 2.2.1 Analytical statistics

Quantitative variables, including age, weight, body mass index (BMI), systolic and diastolic blood pressure, TSH levels, and LT4 dose, were assessed for normality using the Shapiro-Wilk test. Variables following a normal distribution are presented as means ± standard deviations (SD), whereas non-normally distributed variables are reported as medians and interquartile ranges (IQR). Categorical variables, such as sex, comorbidities, food effects, and drug-drug interactions, are summarized using frequencies and percentages.

The relationships between LT4 dose and other quantitative variables were examined using Pearson correlation coefficients for normally distributed data and Spearman rank correlation for non-normally distributed variables.

#### 2.2.2 Variable selection

Univariate analyses, including t-tests, analysis of variance (ANOVA), and chi-square tests, were conducted to identify variables significantly associated with LT4 dose. Stepwise regression (both forward and backward selection) was employed to refine the subset of predictors included in the LT4 dose prediction model, ensuring that only statistically significant and clinically relevant variables were retained.

#### 2.2.3 Model development and optimization

The dataset was randomly divided into a derivation cohort (80%) and a validation cohort (20%) for developing the LT4 dose prediction model. Multiple machine learning algorithms were trained, including XGBoost, Random Forest, LightGBM, Ridge Regression, Lasso Regression, Support Vector Regression (SVR), Decision Tree Regressor (DTR), Gradient Boosting Regressor (GBR), Extra Trees Regressor (ETR), AdaBoost Regressor (ABR), K-Nearest Neighbors Regressor (KNR), Bagging Regressor (BR), Multi-Layer Perceptron Regressor (MLPR), and CatBoost.

Hyperparameter tuning was performed using grid search and cross-validation to optimize model performance. The final hyperparameters were selected based on the best combination of mean absolute error (MAE), mean squared error (MSE), root mean squared error (RMSE), and R² score.

The entire modeling process was implemented using Python (version 11.0), with key libraries including scikit-learn, XGBoost, LightGBM, CatBoost, pandas, numpy, matplotlib, and seaborn. Feature scaling and preprocessing were performed using StandardScaler from scikit-learn, while hyperparameter tuning was conducted using GridSearchCV and RandomizedSearchCV.

#### 2.2.4 Model validation

The final LT4 dose prediction model was validated using 10-fold cross-validation, repeated three times to ensure generalizability and robustness. Validation was conducted in Python using the scikit-learn library, specifically the cross_val_score function. The model’s predictive accuracy was assessed based on the average performance metrics across validation folds, including MAE, MSE, RMSE, and R² score.

The optimized model’s performance before and after hyperparameter tuning was compared using a paired sample t-test to determine statistical significance.

**Figure 1:**
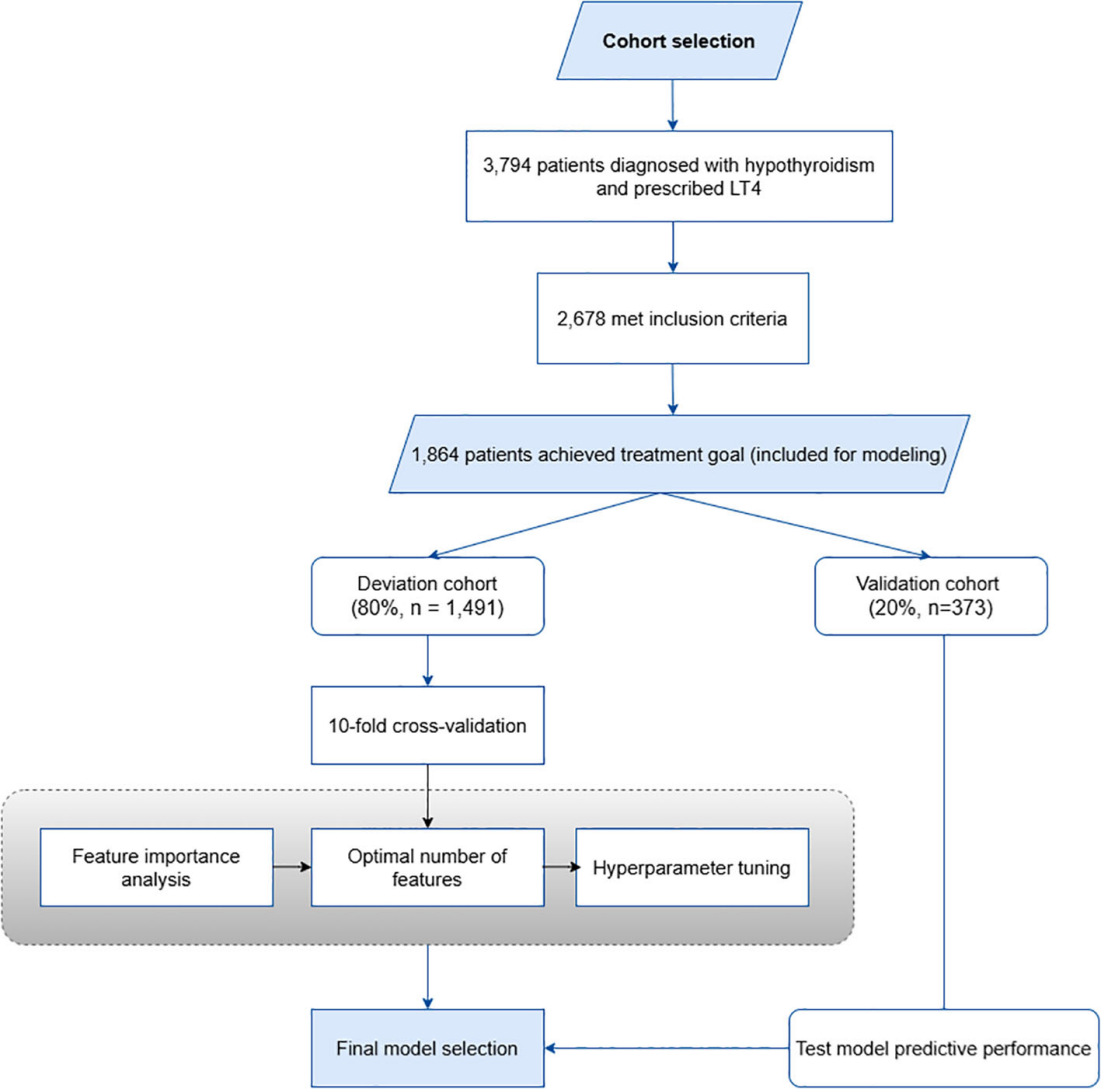
Study flowchart.

The study flowchart is presented in **Figure 1**, and the raw data of the study is provided in **Supplementary Data Sheet 1**.

### 2.3 Ethical considerations

The Institutional Review Board (IRB) of Hai Phong International Hospital, Vietnam, approved and reviewed the study protocols (IRB. 23.118). The study was conducted in accordance with the Declaration of Helsinki and the International Conference on the Harmonization of Technical Requirements for Registration of Pharmaceuticals for Human Use Good Clinical Practice guidelines.

## 3 Results

Of the 3,794 patients diagnosed with hypothyroidism and prescribed LT4, 2,678 met the inclusion criteria. Among the selected patients, 1,864 (approximately 69.6%) met the treatment goal and were included in the analysis and modeling of the LT4 dose calculation (**Figure 1**). The median weight of participants was 54.00 kg (IQR: 47.00–62.43), with a median age of 49.00 years (IQR: 39.00–58.00). The median height was 155.00 cm (IQR: 150.00–160.00), and the median BMI was 22.21 kg/m² (IQR: 19.16–26.59). Blood pressure measurements revealed a median systolic blood pressure (SBP) of 121.00 mmHg (IQR: 110.00–134.00) and a median diastolic blood pressure (DBP) of 72.00 mmHg (IQR: 64.00–80.00). The number of concomitant diseases had a median value of 3.00 (IQR: 2.00–4.00). For patients receiving LT4, the median daily dose was 62.50 mcg (IQR: 50.00–92.80) with individual prescriptions ranging from 25 to 175 mcg. 1,766 (94.74%) patients were prescribed LT4 after meals (**Table 1**).

**Table 1:** Patients’ clinical characteristics (n = 1,864).

Variables	Value (Median (IQR)	Distribution in the dataset (N, %)	Min;Max
Weight (kg)	54.00 (47.00-62.43)		(31;89)
Sex (female)		1703 (91.36%)	
Age (years)	49.00 (39.00-58.00)		(6;86)
Children (6-12)	8.0 (7.0-9.0)	2 (0.11)	(6.0;10.0)
Adolescents (13-18)	17.0 (16.25-18.0)	10 (0.54)	(15.0;18.0)
Young adults (19-44)	35.0 (31.0-41.0)	722 (38.73)	(19.0;44.0)
Middle-aged adults (45-59)	52.0 (48.0-56.0)	723 (38.79)	(45.0;59.0)
Older adults (60-74)	65.0 (62.0-69.0)	381 (20.44)	(60.0;74.0)
Elderly (75-89)	84.0 (78.0-86.0)	26 (1.39)	(76.0;86.0)
Height (cm)	155.00 (150.00-160.00)		(129;185)
BMI	22.21 (19.16-26.59)		(13.24;41.24)
SBP (mmHg)	121.00 (110.00-134.00)		(82;206)
DBP (mmHg)	72.00 (64.00-80.00)		(33;130)
Number of concomitant diseases	3.00 (2.00-4.00)		(1;14)
LT4 dose regimen			
LT4 daily dose (mcg)	62.50 (50.00-92.80)		(25;175)
LT4 taking after meal		1,766 (94.74%)	
Complexity in drug use (ref: complexed)		1,423 (76.34%)	
Comorbidities			
Ulcerative colitis		23 (1.23%)	
*Helicobacter pylori* infection		82 (4.4%)	
Diabetes		423 (22.69%)	
Hypertension		720 (38.63%)	
Hypothyroidism cause			
Post-surgical hypothyroidism		1094 (58.69)	
Post-radioiodine therapy hypothyroidism		264 (14.16)	
Autoimmune hypothyroidism		150 (8.05)	
Drug-induced hypothyroidism		80 (4.29)	
Unknown/Other		276 (14.81)	
Pregnancy		94 (5.04%)	
Concomitant intake of medications			
Calcium supplement		189 (10.14%)	
Iron Preparations		64 (3.43%)	
Magnesium Salts		74 (3.97%)	
Multivitamins		251 (13.47%)	
PPI		134 (7.19%)	
Alendronate		31 (1.66%)	
Potassium chloride		59 (3.17%)	
Ciprofloxacin		4 (0.21%)	
Zinc		6 (0.32%)	
Clinical indices on prescription day			
AST, U/l	23.28 (18.02-30.05)		(10.01;157.13)
ALT, U/l	24.66 (21.22-31.05)		(7.45;306.07)
TSH mIU/l	2.52 (1.50-3.77)		(0.05;6.81)
Serum Albumin (mg/dl)	44.10 (33.23-54.13)		(20.01;64.96)

SBP, systolic blood pressure; DBP: diastolic blood pressure; ALT, alanine aminotransferase; AST, aspartate aminotransferase; BMI, body mass index; SD, standard deviation.

**Figure 2:**
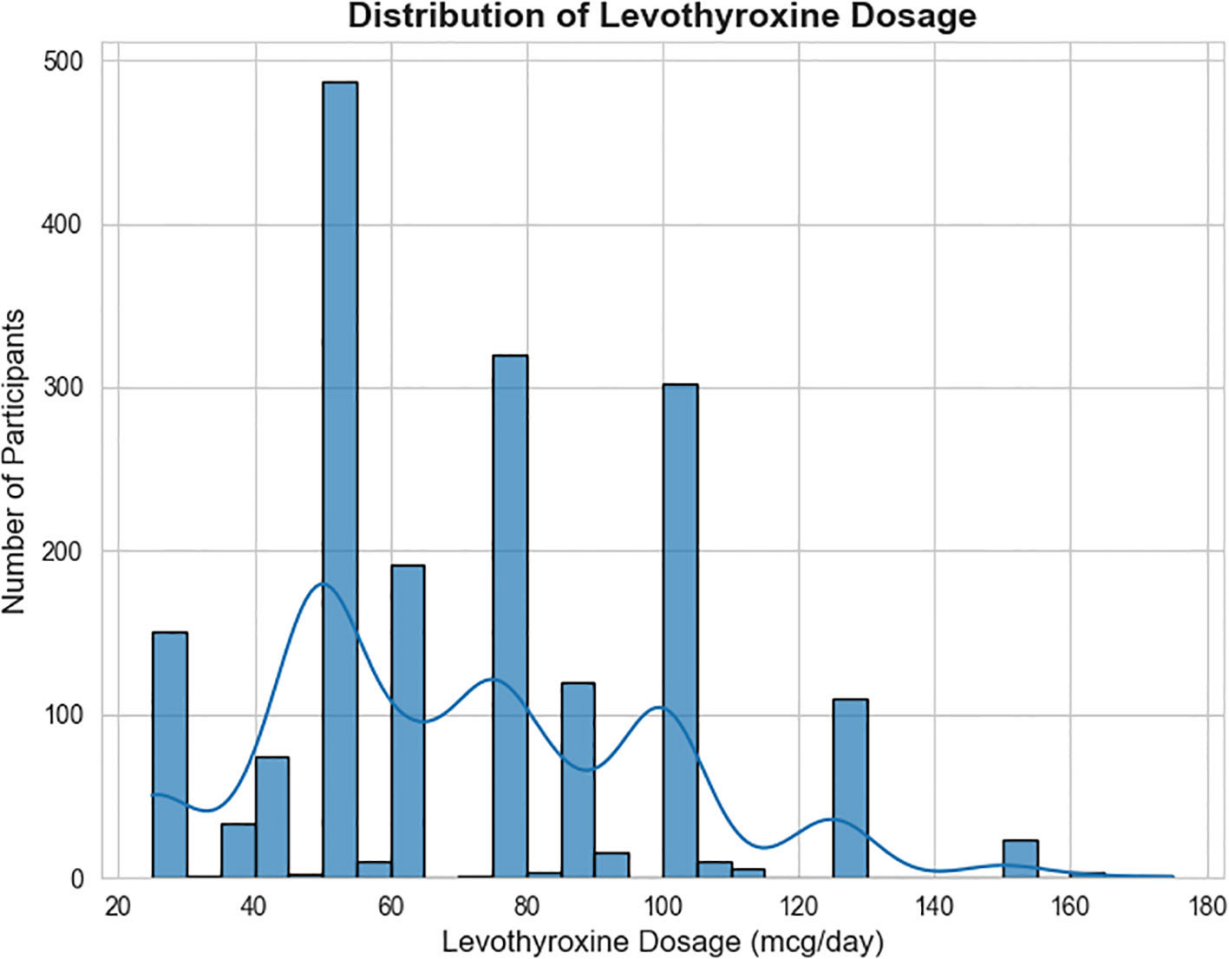
Distribution of Levothyroxine dosage among study participants.

The distribution of LT4 dosage among study participants is visualized in **Figure 2**. The median LT4 dose was 62.5 mcg/day with an interquartile range (IQR) of 50.0 to 92.8 mcg/day. The most commonly prescribed dose was 50.0 mcg/day, accounting for 26.13% of participants. The histogram demonstrates a right-skewed distribution, with most participants receiving doses between 50 to 100 mcg/day. A smaller subset required higher doses (>100 mcg/day), suggesting individualized dosing based on patient-specific factors.

A subanalysis of 1,864 participants revealed that 12 (0.64%) were in the pediatric group (6-18 years) and 1,852 (99.36%) were in the adult group (>18 years). The pediatric group exhibited significantly lower BMI (17.67 vs. 22.26 kg/m², p < 0.001) and LT4 dose requirements (33.75 vs. 62.5 mcg/day, p < 0.001) compared to the adult group. Similarly, GOT (12.12 vs. 23.34 U/L, p < 0.001) and GPT (21.8 vs. 24.7 U/L, p = 0.01) levels were significantly lower in the pediatric group. No significant differences were observed in TSH (2.39 vs. 2.53 mIU/L, p = 0.472) or ALB levels (40.92 vs. 44.1 g/dL, p = 0.362) between the two groups (**Table 2**).

**Table 2:** Comparison of clinical and biochemical parameters between pediatric (6-18 years) and adults (>18 years).

Age Group	Pediatric (6-18)	Adults (>18)	p-value
n (%)	12 (0.64)	1852 (99.36)	
BMI (kg/m²) Median (IQR)	17.67 (15.38-18.47)	22.26 (19.2-26.65)	<0.001
LT4 Dose (mcg/day) Median (IQR)	33.75 (25.0-50.0)	62.5 (50.0-93.0)	<0.001
TSH (mIU/L) Median (IQR)	2.39 (1.68-3.14)	2.53 (1.5-3.78)	0.472
GOT (U/L) Median (IQR)	12.12 (11.7-15.55)	23.34 (18.14-30.11)	<0.001
GPT (U/L) Median (IQR)	21.8 (18.73-23.7)	24.7 (21.25-31.21)	0.01
ALB (g/dL) Median (IQR)	40.92 (29.48-50.21)	44.1 (33.36-54.15)	0.362

The linear regression analysis identified significant predictors of LT4 dosage (**Table 3**). Among demographic factors, BMI (4.657 ± 0.065, P < 0.001) and age (1.013 ± 0.041, P < 0.001) showed strong positive associations with LT4 dosage. Female sex (6.332 ± 2.308, P = 0.006) and pregnancy (18.570 ± 2.938, P < 0.001) were also significant contributors. Comorbidities such as ulcerative colitis (54.456 ± 5.749, P < 0.001), diabetes (21.702 ± 1.467, P < 0.001), Helicobacter pylori infection (40.494 ± 3.026, P < 0.001), and hypertension (13.437 ± 1.298, P < 0.001) were strongly associated with increased LT4 requirements. Regarding hypothyroidism causes, post-surgical hypothyroidism (10.889 ± 1.295, P < 0.001) and post-radioiodine hypothyroidism (4.825 ± 1.860, P = 0.010) were associated with higher LT4 doses, whereas autoimmune (-14.430 ± 2.365, P < 0.001) and drug-induced hypothyroidism (-19.645 ± 3.173, P < 0.001) were linked to lower LT4 requirements. Concomitant medication use significantly influenced LT4 dosing. Proton pump inhibitors (PPIs) (37.162 ± 2.363, P < 0.001), potassium chloride (50.097 ± 3.525, P < 0.001), magnesium salts (43.834 ± 3.169, P < 0.001), iron preparations (43.983 ± 3.419, P < 0.001), calcium supplements (19.294 ± 2.105, P < 0.001), and multivitamins (18.996 ± 1.852, P < 0.001) were associated with significantly increased LT4 requirements. Laboratory findings also showed significant associations. Higher GOT (1.282 ± 0.037, P < 0.001) and GPT (0.701 ± 0.027, P < 0.001) levels were positively correlated with LT4 dosage, while TSH levels after treatment (-2.996 ± 0.406, P < 0.001) were inversely associated with LT4 dosage. Serum albumin was positively associated with LT4 dosage (0.329 ± 0.051, P < 0.001). Other predictors included the number of comorbidities (7.586 ± 0.239, P < 0.001), while taking LT4 before meals (-10.439 ± 2.901, P < 0.001) was associated with a lower LT4 dose requirement.

**Table 3:** Linear regression coefficients for factors influencing LT4 dosage (mcg/day).

Category	Variable	Estimate	Standard Error (SD)	t-value	p-value
**Demographics**	Weight (kg)	1.976	0.039	51.113	< 0.001
	BMI (kg/m²)	4.657	0.065	72.166	< 0.001
	Age (years)	1.013	0.041	24.643	< 0.001
	Female sex	6.332	2.308	2.743	0.006
	Pregnancy	18.57	2.938	6.321	< 0.001
**Comorbidities**	Hypertension	13.437	1.298	10.355	< 0.001
	Diabetes	21.702	1.467	14.789	< 0.001
	Ulcerative colitis	54.456	5.749	9.473	< 0.001
	Helicobacter pylori infection	40.494	3.026	13.382	< 0.001
**Hypothyroidism Cause**	Post-surgical hypothyroidism	10.889	1.295	8.407	< 0.001
	Post-radioiodine hypothyroidism	4.825	1.86	2.594	0.01
	Autoimmune hypothyroidism	-14.43	2.365	-6.102	< 0.001
	Drug-induced hypothyroidism	-19.645	3.173	-6.191	< 0.001
**Concomitant intake of medications**	Proton pump inhibitors	37.162	2.363	15.724	< 0.001
	Potassium chloride	50.097	3.525	14.212	< 0.001
	Magnesium salts	43.834	3.169	13.833	< 0.001
	Iron preparations	43.983	3.419	12.863	< 0.001
	Calcium supplements	19.294	2.105	9.164	< 0.001
	Multivitamins	18.996	1.852	10.258	< 0.001
**Laboratory findings**	Serum albumin (mg/dL)	0.329	0.051	6.398	< 0.001
	GOT (U/L)	1.282	0.037	34.549	< 0.001
	GPT (U/L)	0.701	0.027	26.278	< 0.001
	TSH baseline (mIU/L)	0.251	0.1	2.521	0.012
	TSH after treatment (mIU/L)	-2.996	0.406	-7.388	< 0.001
**Other factors**	Number of comorbidities	7.586	0.239	31.698	< 0.001
	LT4 taken before meals	-10.439	2.901	-3.598	< 0.001

SBP, systolic blood pressure; DBP: diastolic blood pressure; ALT, alanine aminotransferase; AST, aspartate aminotransferase; BMI, body mass index; SD, standard deviation.

**Table 4** compares the performance of various regression models based on MAE with 95% CI and R² values. The ETR achieved the lowest MAE (9.4 [11.2, 7.7]) in the test set and the highest R² (87.37%), making it the best-performing model. Other ensemble methods, including the RFR and GBR, also demonstrated strong predictive accuracy with MAE values around 9.6–9.9 and R² above 80%. Linear regression models (MLR, Ridge, Lasso, LSVR) had significantly higher MAE values (13.5–13.8) and lower R² compared to nonlinear models (p < 0.001 vs. ETR). Among nonlinear models, SVR, NuSVR, and MLPR showed moderate accuracy, while ABR had the highest test set error (12.5 [14.0, 10.9]) with an R² of 80.08%. Overall, tree-based ensemble methods outperformed linear and other nonlinear models, with ETR being the most accurate.

**Table 4:** Comparison of regression models for predicting levothyroxine dosage: mean absolute error (MAE) and R² values.

Regression models	MAE (Training Set) [95% CI]	MAE (Test Set) [95% CI]	R² (Training Set)	R² (Test Set)	P values (vs. MLR)	P values (vs. ETR)
MLR	13.5 [13.6, 13.3]	13.6 [14.7, 12.5]	76.24	74.49	–	< 0.001; < 0.001
RidgeR	13.6 [13.7, 13.4]	13.7 [14.7, 12.6]	76.24	74.5	0.005; 0.695	< 0.001; < 0.001
LassoR	13.5 [13.7, 13.3]	13.6 [14.7, 12.5]	76.11	74.54	0.210; 1.000	< 0.001; < 0.001
LSVR	13.6 [14.1, 13.2]	13.8 [14.7, 12.8]	75.41	75.52	0.023; 0.576	< 0.001; < 0.001
ETR	5.4 [5.6, 5.3]	9.4 [11.2, 7.7]	100	87.37	< 0.001; < 0.001	–
KNR	2.4 [2.5, 2.2]	9.6 [11.6, 7.5]	84.48	78.34	< 0.001; < 0.001	0.743
BR	5.8 [6.0, 5.6]	9.6 [11.7, 7.5]	97.38	83.67	< 0.001; < 0.001	0.681
RFR	6.6 [6.8, 6.4]	9.6 [11.7, 7.7]	98.12	85.39	< 0.001; < 0.001	0.634
XGBR	8.4 [8.6, 8.1]	9.9 [11.2, 8.5]	99.54	84.92	< 0.001; < 0.001	0.234
GBR	7.9 [8.1, 7.8]	9.9 [11.4, 8.4]	89.14	83.55	< 0.001; < 0.001	0.251
DTR	7.3 [7.8, 6.7]	10.1 [12.1, 8.1]	100	71.85	< 0.001; < 0.001	0.142
SVR	7.8 [8.2, 7.3]	10.3 [12.0, 8.7]	43.57	45.68	< 0.001; < 0.001	0.034
NuSVR	8.1 [8.4, 7.9]	10.3 [12.0, 8.6]	43.57	45.68	< 0.001; < 0.001	0.038
MLPR	10.6 [10.9, 10.2]	11.0 [12.5, 9.5]	84.14	78.24	< 0.001; < 0.001	< 0.001
ABR	12.3 [12.4, 12.1]	12.5 [14.0, 10.9]	83.15	80.08	< 0.001; 0.002	< 0.001

MLR, Multiple Linear Regression; RidgeR, Ridge Regression; LassoR, Lasso Regression; LSVR, Linear Support Vector Regression; ETR, Extra Trees Regressor; KNR, K-Nearest Neighbors Regressor; BR, Bagging Regressor; RFR, Random Forest Regressor; XGBR, Extreme Gradient Boosting Regressor; GBR, Gradient Boosting Regressor; DTR, Decision Tree Regressor; SVR, Support Vector Regression; NuSVR, Nu-Support Vector Regression; MLPR, Multilayer Perceptron Regressor; ABR, Adaptive Boosting Regressor.

**Figure 3:**
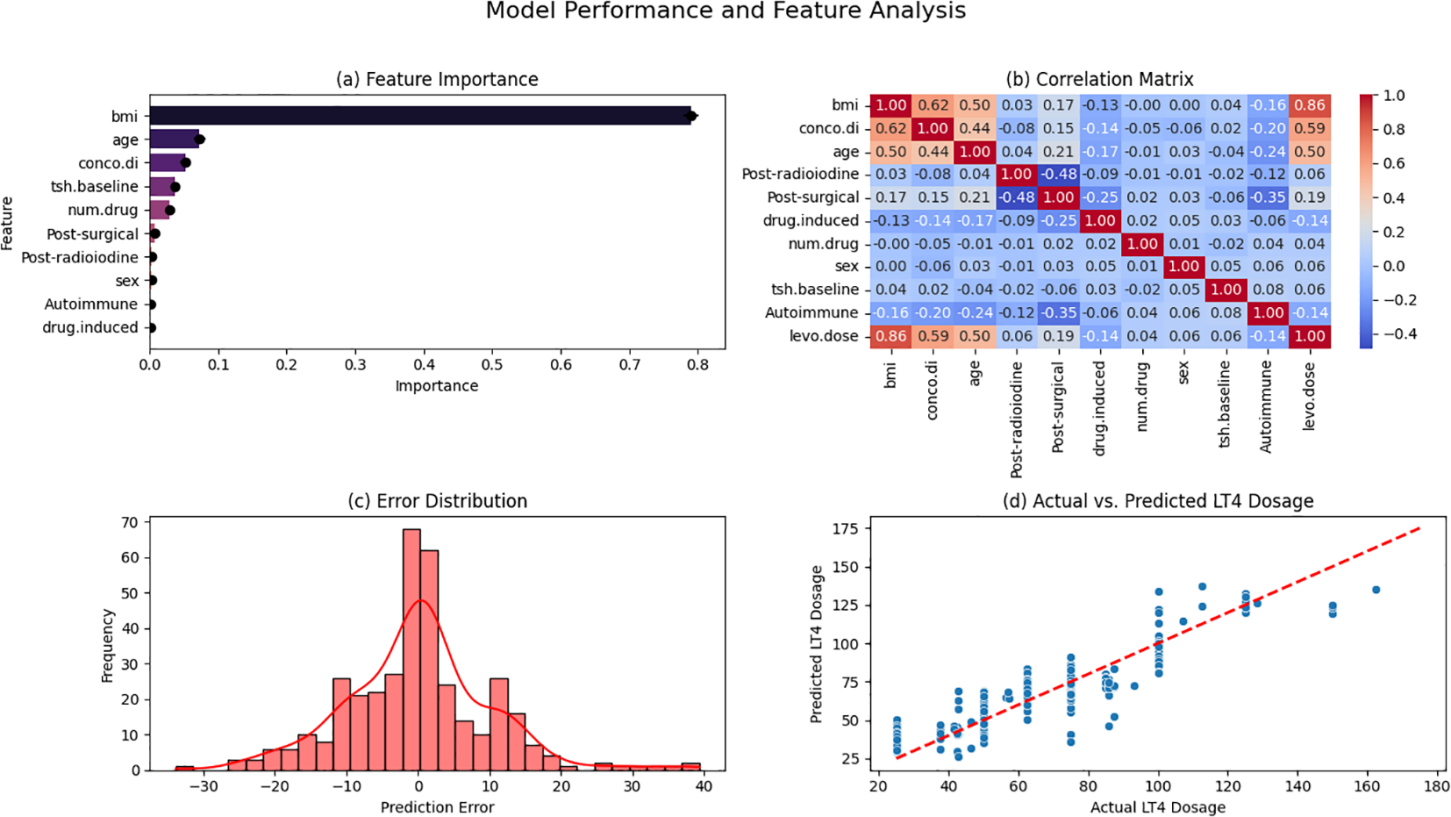
Model performance and feature importance. **(a)** Feature Importance; **(b)** Correlation Matrix; **(c)** Error Distribution; **(d)** Actual vs. Predicted LT4 Dosage. BMI, Body Mass Index; TSH, Thyroid-Stimulating Hormone; LT4, Levothyroxine; Autoimmune, Autoimmune Hypothyroidism; Conco.di, Concomitant Diseases (Comorbidities); Drug.induced, Drug-Induced Hypothyroidism; Num.drug, Total Number of Medications; Post-radioiodine, Post-Radioiodine Therapy; Post-surgical, Post-Surgical Hypothyroidism.

**Figure 3** provides an in-depth analysis of model performance and feature importance in LT4 dose estimation. **Figure 3a** illustrates the relative importance of predictive features, with BMI (0.516 ± 0.015) emerging as the most influential factor, followed by comorbid diseases (0.120 ± 0.010) and age (0.080 ± 0.005). Other significant predictors include post-radioiodine therapy (0.051 ± 0.009), post-surgical hypothyroidism (0.047 ± 0.010), drug-induced hypothyroidism (0.047 ± 0.009), and the number of medications (0.046 ± 0.005), while sex (0.036 ± 0.005), baseline TSH (0.032 ± 0.002), and autoimmune hypothyroidism (0.025 ± 0.007) play a smaller role. **Figure 3b** presents the correlation matrix, highlighting BMI as the strongest predictor of LT4 dosage (r = 0.86, p < 0.001), with age (r = 0.50, p < 0.001) and comorbid diseases (r = 0.59, p < 0.001) also showing moderate correlations. Other variables, including post-radioiodine therapy (r = 0.23, p < 0.001) and post-surgical hypothyroidism (r = 0.19, p < 0.001), exhibit weaker associations, whereas sex (r = 0.06, p = 0.04) and baseline TSH (r = 0.06, p = 0.02) contribute minimally. **Figure 3c** displays the error distribution, showing a near-normal pattern with a mean prediction error of 0.58 (SD = 10.84) and an interquartile range from -7.2 to 8.9, suggesting that most predictions remain within an acceptable range despite occasional outliers. **Figure 3d** presents the actual vs. predicted LT4 dosage, demonstrating a strong alignment along the y = x reference line (R² = 0.85), indicating high predictive accuracy. However, deviations are more pronounced at higher dosages, suggesting areas where model refinements could improve estimation reliability.

## 4 Discussion

This study provides valuable insights into the clinical and biochemical factors influencing LT4 dosage among patients with hypothyroidism. Among the 2,678 participants analyzed, 1,864 individuals (69.6%) achieved treatment goals, forming the basis for dose modeling. The findings underscore the significant variability in LT4 dose requirements, with a median dose of 62.5 mcg/day (IQR: 50.0–92.8 mcg/day) and doses ranging from 25 to 175 mcg/day. These findings align with previous reports that estimate median LT4 dose requirements at 1.3 μg/kg/day (IQR: 0.94,1.60), emphasizing the role of patient-specific metabolic and physiological factors in determining optimal LT4 replacement therapy (34). Our analysis highlights key predictors of LT4 dosage, which are consistent with findings from previous studies. BMI emerged as the strongest predictor, with a positive association (4.657 ± 0.065 mcg/kg/m², p < 0.001). The majority of patients were female (1,703; 91.36%). Research findings consistently indicate a higher prevalence of hypothyroidism in women compared to men. Reports suggest that hypothyroidism is approximately 4-6 times more prevalent in women than in men (35). However, the underlying reasons for this gender disparity remain incompletely understood. Some studies propose hormonal factors, such as estrogen, as potential contributors to the higher prevalence of hypothyroidism in women, given their influence on thyroid function. Other research suggests lifestyle factors, including diet and physical activity, may also influence the gender difference (36). Notably, the prevalence of hypothyroidism varies across diverse populations and regions, emphasizing the need for further investigation to gain a comprehensive understanding of gender differences in hypothyroidism prevalence.

Our analysis highlights BMI as a key predictor of LT4 dosage, consistent with previous studies emphasizing its role in thyroid hormone replacement therapy. In our study, BMI demonstrated a strong positive association with LT4 dose (4.657 ± 0.065 mcg/kg/m², p < 0.001), reinforcing the metabolic demands of patients with higher body mass. This aligns with prior research indicating that weight-based dosing alone may be insufficient for achieving euthyroidism, particularly in overweight and underweight individuals (28, 37). Studies have shown that patients with higher BMI require higher absolute LT4 doses but lower doses relative to body weight, suggesting a complex interplay between body composition and thyroid hormone metabolism (38, 39). While BMI-based dosing algorithms have been developed and have improved euthyroidism rates at follow-up, recent studies advocate for more sophisticated dosing models incorporating multiple clinical parameters (40). Notably, some researchers argue that TSH, body weight, and BMI alone may be insufficient for precise LT4 titration, highlighting the need for individualized, multifactorial approaches to therapy optimization (28).

As per the investigation, the mean age of the patients was 48.42 ± 13.72, ranging from 6 to 86 years old. The prevalence of hypothyroidism varies across age groups. According to previous research, the highest prevalence of hypothyroidism is observed in older people (41). This study highlights significant differences in clinical and biochemical parameters between pediatric and adult patients on LT4 therapy, consistent with findings in existing literature. Pediatric patients often require higher weight-based LT4 doses compared to adults (42), likely due to age-related variations in thyroid hormone metabolism and persistent hypothalamic-pituitary resistance in congenital cases. The lower BMI (17.67 vs. 22.26 kg/m², p < 0.001) and LT4 dose requirements (33.75 vs. 62.5 mcg/day, p < 0.001) observed in the pediatric group reflect differences in body composition and metabolic rates. Developmental variations in liver enzyme activity may explain the significantly lower GOT (12.12 vs. 23.34 U/L, p < 0.001) and GPT (21.8 vs. 24.7 U/L, p = 0.01) levels in pediatric patients, as children are known to metabolize LT4 differently, potentially leading to increased reactive metabolite formation (43). Despite these differences, comparable TSH levels (2.39 vs. 2.53 mIU/L, p = 0.472) across groups suggest effective dose titration strategies, aligning with the principle that age-specific reference intervals for thyroid function tests may be necessary (44). The small sample size in the pediatric group is a limitation of this study. Further research with larger cohorts is needed to validate these findings and refine LT4 dosing strategies, particularly for pediatric patients, to mitigate risks such as iatrogenic hyperthyroidism observed in initial dosing (45). Age-specific approaches remain critical to optimizing thyroid hormone replacement therapy and preventing overtreatment or undertreatment in diverse populations.

In our participants, we observed several notable comorbidities and conditions that warrant attention in the management of LT4 dose. Among patients with hypothyroidism, a substantial proportion (52.58%) underwent thyroidectomy, indicating a significant prevalence of surgical interventions in this population. A novel Poisson regression model incorporating seven variables predicted 60.9% of the doses (p=0.031) based on the data of 598 patients who attained euthyroidism after total or complete thyroidectomy for benign diseases (46). Furthermore, hypertension emerged as the most prevalent comorbidity among patients with hypothyroidism, affecting 38.63% of the individuals in our study cohort. Diabetes mellitus was also observed to be prevalent among hypothyroid patients, affecting 22.69% of the individuals. This finding underscores the bidirectional relationship between thyroid dysfunction and metabolic disorders and emphasizes the need for integrated management strategies to address both conditions simultaneously. Interestingly, adenocarcinoma of the thyroid gland was identified in 29.51% of patients with hypothyroidism, indicating a notable prevalence of thyroid malignancies within this population.

In addition to these common comorbidities, this study investigated the prevalence of gastrointestinal diseases in patients with hypothyroidism. Notably, a significant proportion of patients receiving LT4 (LT4) treatment present with concurrent conditions that can potentially impair intestinal absorption of the medication. Conditions such as gastroesophageal reflux disease (GERD), irritable bowel syndrome (IBS), food allergies, lactose intolerance, gastric bypass, Helicobacter pylori infection, gastroparesis, celiac disease, ulcerative colitis, Crohn’s disease, and atrophic gastritis can all interfere with the absorption of LT4, thus affecting treatment efficacy and patient outcomes (47, 48). In our study, ulcerative colitis was identified in 1.23% of patients, while Helicobacter pylori infection was observed in 4.4%. Ulcerative colitis can significantly affect LT4 absorption, leading to an increased need for the medication in hypothyroid patients. Studies have shown that ulcerative colitis patients require approximately 26% higher LT4 doses compared to controls to achieve similar TSH levels (47). This malabsorption may persist even during clinical remission of UC. Various gastrointestinal disorders, including inflammatory bowel diseases, can impede LT4 absorption (7, 49). The development of new oral formulations, such as liquid LT4, may help overcome malabsorption issues in patients with gastrointestinal disorders (50). Patients requiring unusually high LT4 doses should be evaluated for potential malabsorption conditions (51). The LT4 absorption test can be useful in distinguishing between true malabsorption and pseudomalabsorption (52). Additionally, Ulcerative colitis may affect bile acid absorption, which could further impact medication absorption (53). Our study also included a subgroup analysis focused on pregnant women in the study cohort. Pregnancy was associated with markedly higher LT4 requirements (18.570 ± 2.938 mcg, p < 0.001), consistent with studies demonstrating that thyroid hormone demand increases significantly during gestation to maintain maternal and fetal euthyroidism (54, 55). This heightened demand is driven by increased thyroid-binding globulin levels, hCG stimulation of TSH receptors, and elevated peripheral thyroid hormone metabolism (56). Our findings align with previous reports indicating that 84–85% of hypothyroid women require LT4 dose increases during pregnancy, typically within the first 5–7 weeks of gestation (57). These results underscore the critical importance of early LT4 dose adjustments, particularly in the first trimester, to maintain optimal thyroid function. While trimester-specific TSH targets are widely used, clinical approaches to screening and dose adjustment vary across regions, particularly in Europe and Asia (58, 59).

In our study, we also assessed the effect of concurrent medications along with LT4. These included calcium supplements (10.14%), iron preparations (3.43%), magnesium salts (3.97%), multivitamins (13.47%), proton pump inhibitors (PPIs) (7.19%), alendronate (1.66%), potassium chloride (3.17%), ciprofloxacin (0.21%), and zinc supplements (0.32%). Notably, all these medications have been documented to potentially influence the pharmacokinetics or dosage requirements of LT4. Calcium supplements, for instance, may interfere with LT4 absorption when administered simultaneously, whereas iron preparations can bind to LT4 in the gastrointestinal tract, reducing its absorption (60). Similarly, magnesium salts and PPIs are associated with decreased LT4 absorption, potentially necessitating dosage adjustments. Multivitamins containing iron or calcium, such as alendronate, potassium chloride, and ciprofloxacin, may also affect LT4 absorption. Zinc supplementation may also interfere with LT4 absorption, albeit to a lesser extent (61). Our findings are consistent with the existing literature regarding the influence of these medications on the daily dosage requirements of LT4, except ciprofloxacin and zinc. However, ciprofloxacin and zinc did not appear to significantly affect the daily LT4 dosage requirements in our study. While these medications have been reported to interact with LT4 in some contexts, our findings suggest that their impact on LT4 dosage may be minimal or negligible within our study population (62, 63).

In our study, we found an influence of liver function (AST and ALT levels) and serum albumin levels on LT4 dosage. These parameters are of particular interest owing to their potential roles in thyroid hormone metabolism and distribution within the body. The liver plays a crucial role in the metabolism and clearance of thyroid hormones, including LT4. Impaired liver function can affect the synthesis of binding proteins such as TBG, which may in turn influence the distribution and availability of circulating thyroid hormones (64). Liver dysfunction can alter the metabolism of medications and potentially affect LT4 pharmacokinetics. Similarly, the serum albumin level is an important determinant of thyroid hormone transport and distribution. Albumin serves as the major carrier protein for thyroid hormones in the bloodstream, facilitating their transport to target tissues. Changes in albumin levels can affect the binding capacity of thyroid hormones and alter their distribution and availability for cellular uptake (65).

Our study confirms the effectiveness of machine learning models in predicting LT4 dosage with high accuracy, aligning with previous research. Among the tested models, ensemble methods such as Extra Trees Regressor (ETR) and Random Forest demonstrated superior performance, with ETR achieving the highest R² (0.87) and the lowest mean absolute error (MAE) in the test set. These findings are consistent with prior studies where XGBoost outperformed standard dosing guidelines by integrating a broader range of clinical variables beyond BMI and age (66). Additionally, our results highlight the significance of diverse predictors, including pregnancy, comorbidities, and concomitant medications, in determining LT4 requirements. This aligns with Singh et al., who employed multivariable linear regression for LT4 dose prediction, achieving early euthyroidism in 68% of participants (67, 68). Similar approaches were observed in the studies by Liu et al. and Zaborek et al., where machine learning models consistently outperformed conventional weight-based dosing methods. Notably, Zaborek et al. identified Poisson regression as the most accurate model among 13 machine learning algorithms (46). Collectively, these findings suggest that integrating machine learning into clinical practice can improve personalized LT4 dosing, enhancing treatment outcomes for hypothyroid patients.

The machine learning-based LT4 dosing model developed in this study offers a precise, data-driven approach to optimizing hypothyroidism treatment. By integrating key clinical variables—including BMI, age, sex, comorbidities, drug interactions, and biochemical markers—our model outperforms traditional weight-based dosing strategies, reducing variability and improving dose accuracy. Feature selection ensured clinical relevance, with ETR achieving the lowest MAE (9.4 [11.2, 7.7]) and highest R² (87.37%), demonstrating superior predictive performance. The low MAE highlights the model’s precision in capturing individual LT4 requirements, reducing the need for trial-and-error adjustments and facilitating faster achievement of target TSH levels. This approach could streamline clinical workflows, minimize unnecessary follow-ups, and enhance treatment outcomes. The model’s integration into clinical decision support systems or electronic health records could provide real-time, personalized dosing recommendations, improving efficiency in hypothyroidism management. Further validation in external cohorts and prospective trials is needed to confirm its real-world applicability.

One limitation of this study is its retrospective design, which relies on data collected from electronic medical records. Retrospective studies are prone to inherent biases and limitations, such as incomplete or missing data, variable data quality, and the inability to establish causality. Additionally, this study did not account for patient adherence to LT4 therapy, a critical factor influencing treatment efficacy. Non-adherence to LT4 is a well-recognized issue that can lead to fluctuations in thyroid hormone levels, suboptimal symptom control, and potential misinterpretation of dosage requirements. Variability in adherence may also contribute to differences in biochemical parameters observed between pediatric and adult patients. Future prospective studies incorporating objective measures of adherence, such as pharmacy refill data or serum LT4 monitoring, along with long-term treatment outcomes and quality-of-life assessments, are needed to refine LT4 dosing strategies and optimize management for individuals with hypothyroidism.

## 5 Conclusions

In conclusion, we comprehensively examined both clinical and non-clinical variables, uncovering significant relationships with numerous factors, such as weight, sex, age, BMI, diastolic blood pressure, comorbidities, food effect, drug-drug interactions, liver function, serum albumin, and TSH levels. Machine learning models were developed to predict LT4 dosage, with the Extra Trees Regressor demonstrating the best performance (MAE: 9.4 [11.2, 7.7], R²: 87.37%), outperforming linear and other nonlinear models. BMI was the strongest predictor of LT4 dosage, followed by comorbid conditions and age. Additional significant factors included post-radioiodine therapy, post-surgical hypothyroidism, drug-induced hypothyroidism, and medication use. Correlation analysis confirmed BMI (r = 0.86, p < 0.001) as the primary determinant. Model residuals showed a near-normal distribution, and actual vs. predicted dosage plots demonstrated high predictive accuracy. These findings highlight the potential of machine learning in individualized LT4 dose optimization, improving precision in hypothyroidism management. Further validation is necessary for clinical application.

## Data Availability

The raw data supporting the conclusions of this article will be made available by the authors, without undue reservation.
